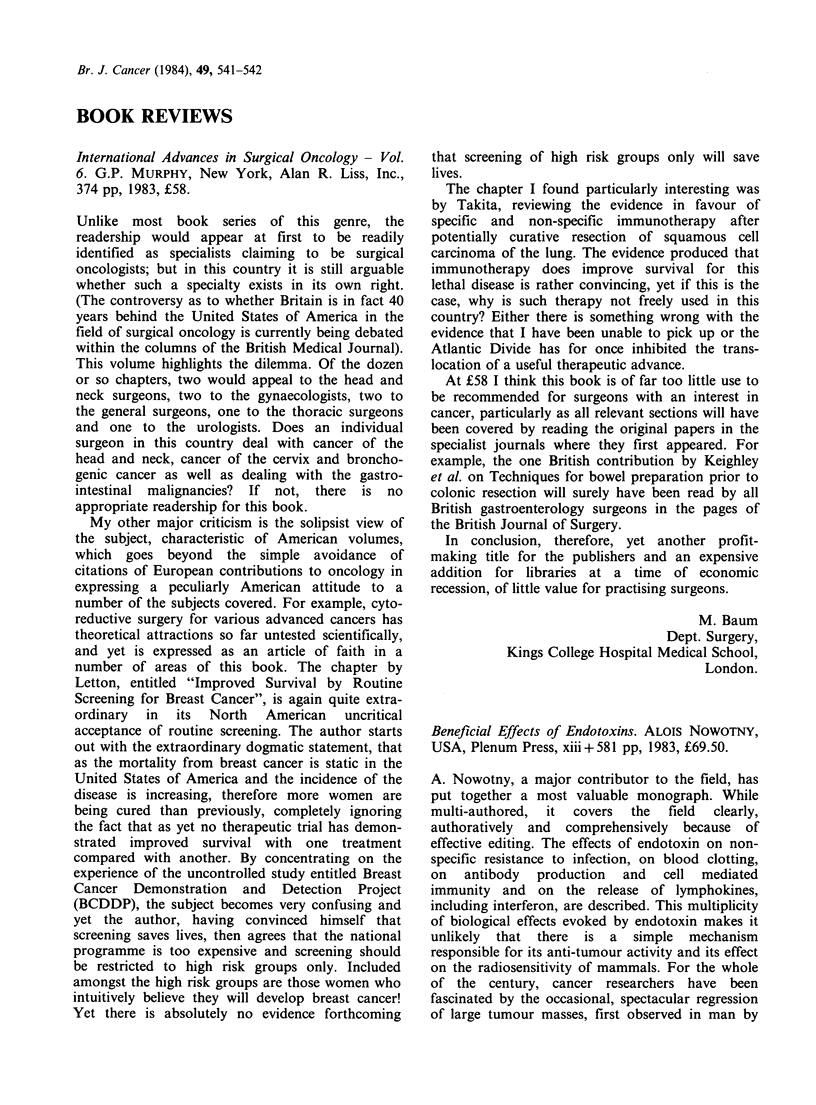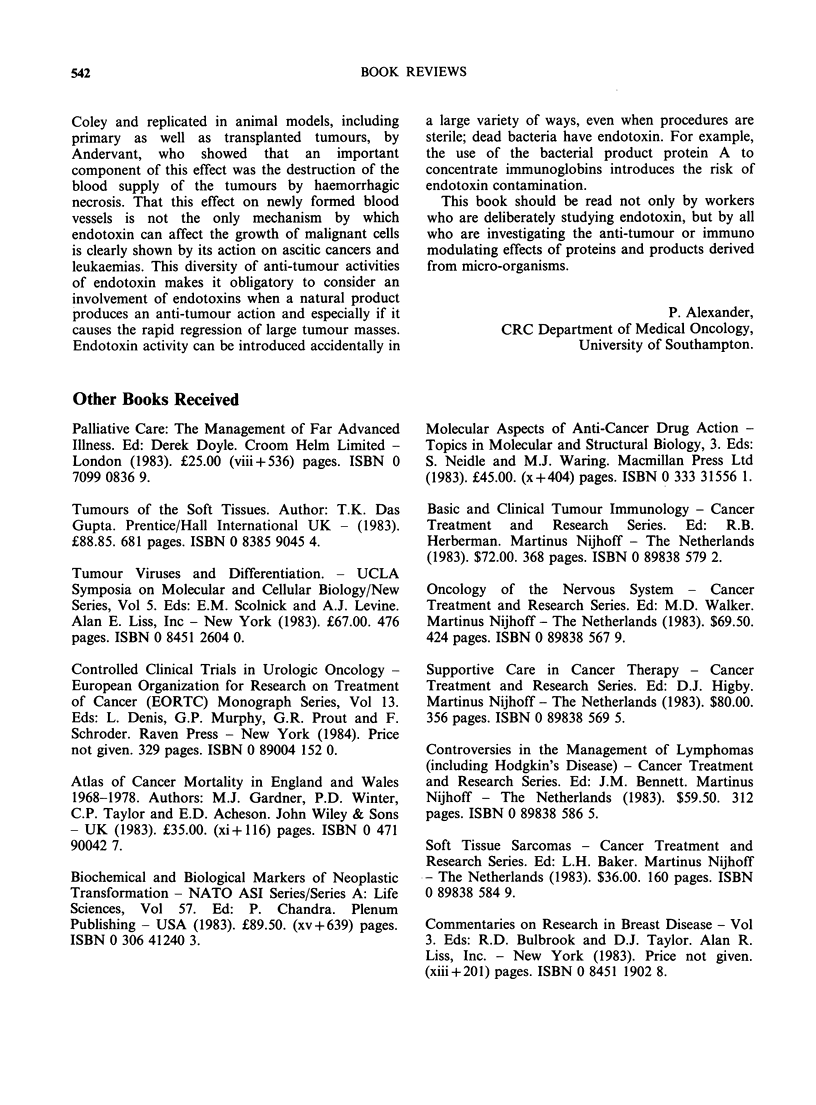# Beneficial Effects of Endotoxins

**Published:** 1984-04

**Authors:** P. Alexander


*Beneficial Effects of Endotoxins*. ALOIS NOWOTNY, USA, Plenum Press, xiii + 581 pp, 1983, £69.50.

A. Nowotny, a major contributor to the field, has put together a most valuable monograph. While multi-authored, it covers the field clearly, authoratively and comprehensively because of effective editing. The effects of endotoxin on non-specific resistance to infection, on blood clotting, on antibody production and cell mediated immunity and on the release of lymphokines, including interferon, are described. This multiplicity of biological effects evoked by endotoxin makes it unlikely that there is a simple mechanism responsible for its anti-tumour activity and its effect on the radiosensitivity of mammals. For the whole of the century, cancer researchers have been fascinated by the occasional, spectacular regression of large tumour masses, first observed in man by

Coley and replicated in animal models, including primary as well as transplanted tumours, by Andervant, who showed that an important component of this effect was the destruction of the blood supply of the tumours by haemorrhagic necrosis. That this effect on newly formed blood vessels is not the only mechanism by which endotoxin can affect the growth of malignant cells is clearly shown by its action on ascitic cancers and leukaemias. This diversity of anti-tumour activities of endotoxin makes it obligatory to consider an involvement of endotoxins when a natural product produces an anti-tumour action and especially if it causes the rapid regression of large tumour masses. Endotoxin activity can be introduced accidentally in a large variety of ways, even when procedures are sterile; dead bacteria have endotoxin. For example, the use of the bacterial product protein A to concentrate immunoglobins introduces the risk of endotoxin contamination.

This book should be read not only by workers who are deliberately studying endotoxin, but by all who are investigating the anti-tumour or immuno modulating effects of proteins and products derived from micro-organisms.

P. Alexander,
CRC Department of Medical Oncology,
University of Southampton.